# Neural Research on Depth Perception and Stereoscopic Visual Fatigue in Virtual Reality

**DOI:** 10.3390/brainsci12091231

**Published:** 2022-09-11

**Authors:** Mei Guo, Kang Yue, Haochen Hu, Kai Lu, Yu Han, Shanshan Chen, Yue Liu

**Affiliations:** 1 Beijing Engineering Research Center of Mixed Reality and Advanced Display, School of Optics and Photonics, Beijing Institute of Technology, Beijing 100081, China; 2 Institute of Software, Chinese Academy of Sciences, Beijing 100045, China

**Keywords:** binocular disparity, stereoscopic visual fatigue, virtual reality, visual evoked potentials

## Abstract

Virtual reality (VR) technology provides highly immersive depth perception experiences; nevertheless, stereoscopic visual fatigue (SVF) has become an important factor currently hindering the development of VR applications. However, there is scant research on the underlying neural mechanism of SVF, especially those induced by VR displays, which need further research. In this paper, a Go/NoGo paradigm based on disparity variations is proposed to induce SVF associated with depth perception, and the underlying neural mechanism of SVF in a VR environment was investigated. The effects of disparity variations as well as SVF on the temporal characteristics of visual evoked potentials (VEPs) were explored. Point-by-point permutation statistical with repeated measures ANOVA results revealed that the amplitudes and latencies of the posterior VEP component P2 were modulated by disparities, and posterior P2 amplitudes were modulated differently by SVF in different depth perception situations. Cortical source localization analysis was performed to explore the original cortex areas related to certain fatigue levels and disparities, and the results showed that posterior P2 generated from the precuneus could represent depth perception in binocular vision, and therefore could be performed to distinguish SVF induced by disparity variations. Our findings could help to extend an understanding of the neural mechanisms underlying depth perception and SVF as well as providing beneficial information for improving the visual experience in VR applications.

## 1. Introduction

Stereoscopic visual fatigue (SVF) is an important problem hindering the development of stereoscopic display applications, especially virtual reality (VR) display applications for deep immersion. SVF, which can be manifested as blurred vision, diplopia and other binocular anomalies symptoms, is a state of weakness, easy fatigue, and unsustainable vision of a visual-related organ that occurs after viewing the stereoscopic content due to excessive load [1,2,3,4]. The long-term exposure to electronics-induced visual fatigue is associated with a variety of serious visual health problems, such as decreased retinal vision, optic neurasthenia, dry eyes, cataracts, and glaucoma [5,6,7]. Therefore, it is important to assess SVF at an early stage before visual health deteriorates and to improve the stereoscopic display technology for comfortable viewing. Previous literature revealed that the vergence–accommodation conflict (VAC) and excessive binocular disparity were the main causes of SVF induced by parallax stereoscopic displays [3,8,9,10,11]. However, the evaluation method and underlying neural mechanism of SVF, especially those induced by VR displays, still need further research.

Visual evoked potentials (VEPs) are typically extracted from scalp-recorded electroencephalography (EEG) through signal averaging and are linked in time with a specific visual sensory and perception event, which are one of the most informative and dynamic methods for monitoring the brain information stream in real time [12,13]. Thus, VEPs are becoming one of the most effective methods to investigate the human visual cognitive function in recent years and are expected to be used as an objective measure of response to a visual stimulus [14,15,16]. Typical VEP components, such as P1 (120 ms), N1 (170 ms), and P2 (300 ms), can be utilized as indicators to reflect a certain process of visual information perception, and therefore changes in features of VEP components may suggest functional changes in certain brain areas [17,18,19,20,21,22,23]. Disparity variations have been adopted to evoke VEPs [24]. Random dot stereograms (RDSs) which portray areas pulsating in depth, have proved to be useful stimuli in the study of human stereopsis, as well as being obvious candidates for probing VEPs [25].

To investigate the neural mechanism of SVF, an experimental paradigm based on the Go/NoGo task was designed, and the experimental scene was constructed and presented by a head-mounted display (HMD). SVF in the experiment was induced by disparity variations with VAC. The Go/NoGo paradigm is usually adopted to measure participants’ capacity for sustained attention and response control [26,27], and it is therefore designed to maintain subjects’ attention levels during the SVF experiment. RDSs were presented as visual stimuli to separate the disparity variations, thereby allowing the time-domain features of VEPs caused by disparity variations to be obtained from the subsequent analysis. Point-by-point statistics [28] and one-way repeated-measures analysis of variance (ANOVA) were used to explore the relationship between the disparities and characteristics of VEP components as well as the relationship between SVF and characteristics of VEP components. The main contributions of this paper are as follows: (1) Modified attention maintained VEP paradigm was proposed in VR environment, in which a uniform reference frame was designed to extract VEPs characteristics evoked by relative disparities. (2) Posterior P2 of VEPs was reported to be associated with depth perception and SVF. (3) The location of posterior component P2 related to depth perception and SVF was obtained by source localization.

## 2. Materials and Methods

### 2.1. Participants

A total of 14 right-handed healthy adults (aged 24±1.1 years, 6 males and 8 females) were recruited from a cohort of graduate students at Beijing Institute of Technology (Beijing, China) to participate in the study. Participants had normal or corrected-to-normal vision with normal stereoscopic visual senses, no degenerative, neurological, psychiatric conditions known to affect cognition, and no visual or vestibular disorders known to affect visual function (self-reported). Written informed consent was obtained from each participant.

### 2.2. Experimental Environment

**Figure 1:**
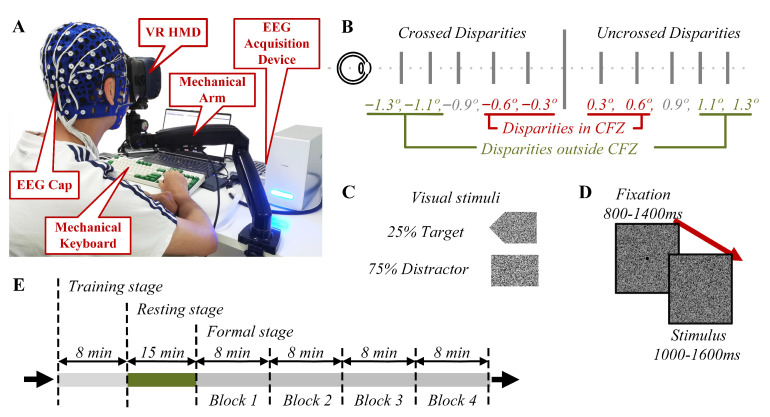
Schematic of the experimental environment, stimuli, trials and procedure. (**A**) Experimental environment. (**B**) Disparity design. Ten disparity settings in the experiment, with $-0.3^{\circ }$, $-0.6^{\circ }$, $-0.9^{\circ }$, $-1.1^{\circ }$, $-1.3^{\circ }$ for the crossed disparities, $0.3^{\circ }$, $0.6^{\circ }$, $0.9^{\circ }$, $1.1^{\circ }$, $1.3^{\circ }$ for the uncrossed disparities, $-0.6^{\circ }$, $-0.3^{\circ }$, $0.3^{\circ }$, $0.6^{\circ }$ for the disparities in comfort fusion zone, $-1.3^{\circ }$, $-1.1^{\circ }$, $1.1^{\circ }$, $1.3^{\circ }$ for the disparities outside comfort fusion zone. (**C**) Visual stimuli. The stimulus was divided into arrow and square shapes. The arrow stimulus was the target stimulus with a 25% occurrence probability, and the square stimulus was the interference stimulus for the Go/Nogo task with a 75% occurrence probability. (**D**) Diagram of a single trial. The duration of $0^{\circ }$ disparity planes as fixation reference was 800–1400 ms, and the presentation duration of the subsequent stimulus was 1000–1600 ms. (**E**) Experimental procedure. Three consecutive stages were performed in this experiment.

The experimental environment is shown in Figure 1A. Participants were seated on a comfortable, height-adjustable chair in a quiet room with good air condition. An HTC Vive Pro head-mounted display (HMD) was used as the display unit, which features dual displays (one per eye) and each display is a 3.5-inch AMOLED with a resolution of 1440×1600 pixels. The refresh rate is 90 Hz, and the horizontal field of view (FOV) of the HMD is $110^{\circ }$. Participants were asked to wear an EEG cap from Compumedics NeuroScan with 64 electrodes during the experiment. In order to reduce interference during EEG collection, the HMD was fixed on an adjustable mechanical arm instead of being worn on the participants’ head.

### 2.3. Stimuli and Procedure

The stimulating scenes and experimental tasks were designed on the basis of a disparity-based Go/NoGo paradigm and were presented to participants using Unity 3D (Unity Technologies, San Francisco, CA, USA). The experimental stimuli were designed as RDSs to induce SVF with a dot density of 50 dot/deg${}^{2}$. The background of the scene was set to all black to avoid the interference of ambient light brightness. Stimuli shapes square or arrow($4.03^{\circ }\times 2.69^{\circ }$) of the left and right views of RDS located at central areas of the random dots plane ($38.82^{\circ }\times 42.76^{\circ }$) were shifted horizontally inwards or outwards to form crossed or uncrossed disparities.

As shown in Figure 1B, the shifts had 10 angular disparities ($-1.3^{\circ }$, $-1.1^{\circ }$, $-0.9^{\circ }$, $-0.6^{\circ }$, $-0.3^{\circ }$, $0.3^{\circ }$, $0.6^{\circ }$, $0.9^{\circ }$, $1.1^{\circ }$, $1.3^{\circ }$) on the subject’s retina when the RDSs were displayed at a horizontal depth of 613 mm from the subjects in the virtual environment. Stimuli shapes of arrow and square (see Figure 1C) were set to perform the Go/NoGo task with a proportion of 1:3, in which the participants were asked to press either up-direction or down-direction button whenever they saw the arrow shape behind or in front of the background plane clearly as soon as possible in order to maintain attention and keep the task performance on a desired level. The single trial structure is shown in Figure 1D. In order to evaluate disparities with a consistent standard, each disparity change was transformed from the $0^{\circ }$ disparity plane to the stimulus presentation disparity plane. A black point (0.1 degree radius) superimposed onto the middle of the pattern helped the subjects to fixate their eyes and prevented voluntary eye movements. Moreover, in order to avoid the expected response to the stimuli, the presentation time of $0^{\circ }$ disparity planes as fixation reference was set to 800–1400 ms in random, and the subsequent stimulus, which was alternately presented to evoke VEPs [24], was set to 1000–1600 ms, randomly.

The experimental task was performed in three consecutive stages as shown in Figure 1E. In the first stage (i.e., training stage), subjects were asked to conduct an 8 min Go/NoGo session to become familiarized with the experimental process to minimize the practice effect of the experimental task, as well as to ensure that all disparities could be seen clearly. In the second stage (i.e., resting stage), participants took a 15 min break to relax and to ensure a comfortable state, then they were asked to score their visual fatigue status on a Likert 5-scale, for which 1 point represents no visual fatigue at all and 5 points represent very severe visual fatigue. In the third stage (i.e., formal stage), simultaneous measurement was performed and whole-scalp EEG data were recorded while the participant was completing the viewing task for a total duration of nearly 32 min. The third stage consisted of four repetitive blocks, and each lasted 8 min. The inducement of SVF was confirmed by the scoring of the subjective visual fatigue status right after the viewing task of each block, of which the evaluation standard is the same as that in the resting stage. Stimulus trials were presented randomly in each block with equal number of presentations for each disparity. Each block contained 50 target stimulus trials and 150 distractor stimulus trials. The target stimulus was presented to maintain the attention of participants and the distractor trial-evoked VEPs for subsequent EEG analysis.

### 2.4. EEG Acquisition

**Figure 2:**
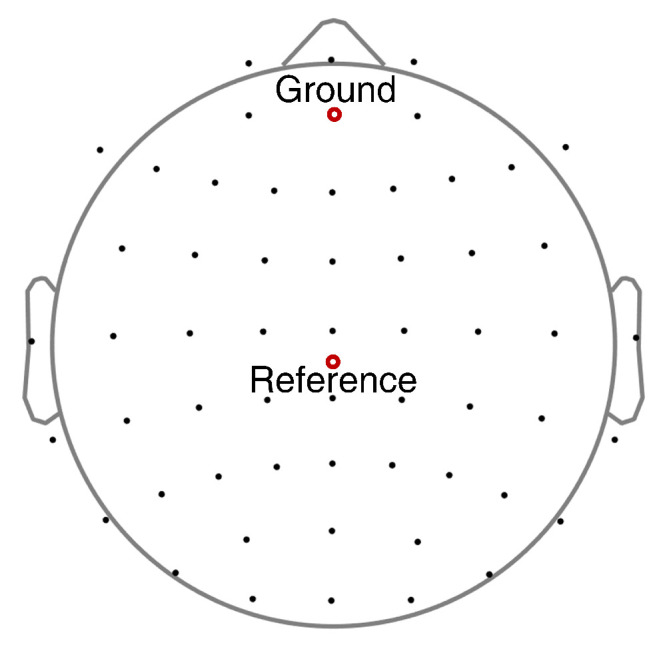
EEG signals were recorded according to the international 10–20 system.

EEG data were collected through the Compumedics NeuroScan SynAmps2 64-channel amplifier head-box. All channels were placed according to the international 10–20 system and covered the whole brain regions. Fifty-six electrodes (FP1, FP2, FPz, AF3, AF4, F1, F2, F3, F4, F5, F6, F7, F8, Fz, FC1, FC2, FC3, FC4, FC5, FC6, FCz, C1, C2, C3, C4, C5, C6, T7, T8, Cz, CP1, CP2, CP3, CP4, CP5, CP6, TP7, TP8, CPz, P1, P2, P3, P4, P5, P6, P7, P8, Pz, PO3, PO4, PO7, PO8, POz, O1, O2, Oz) were used for recording the EEG activities within the scope of this study. The reference and ground electrodes of all scalp channels are shown in Figure 2. EEG data were digitized with a 24-bit analog-to-digital converter with a sampling rate of 1000 Hz. The electrode impedance at each electrode was maintained below 10 kΩ.

### 2.5. EEG Data Analysis

EEG data processing and analysis were performed offline with EEGLAB Toolbox version 20 [29].

#### 2.5.1. EEG Data Pre-Processing

The raw EEG data from NoGo trials of all four blocks were attached to create a dataset for each subject and then resampled to 250 Hz to reduce the computational requirements for further processing steps. The resampled EEG data were bandpass filtered into a 0.1 to 40 Hz frequency range with a Parks McClellan notch filter at 50 Hz in the meantime and then digitally re-referenced to the common average reference. In addition, artifact subspace reconstruction (ASR), i.e., a plug-in of the EEGLAB, was applied with the threshold σ=20 to reduce data contamination by high variance artifacts [30]. Thereafter, the trial epochs lasted 1000 ms, with a 200 ms pre-stimulus baseline, which was extracted from the continuous EEG data. The adaptive independent component analysis mixture model algorithm (AMICA), which is a generalization of the infomax algorithm and multiple mixture independent component analysis approaches, was applied to the epoch data [31]. Typical artifacts, such as eye movement, blink and muscle artifacts, produced a certain pattern in the EEG data and can be separated by AMICA into a few independent components (ICs), which were visually inspected considering the power spectrum and event-locked time course to obtain the separation of ICs associated with brain activity. Then, the artifact ICs were removed to form the clean EEG epoch data.

#### 2.5.2. Time-Domain Analysis of VEPs

Time-domain analysis of VEPs was performed with the STUDY module of EEGLAB among experimental factors, i.e., disparities and blocks. In our study, EEG data collected from electrodes F3, F1, Fz, F2, F4, FC3, FC1, FCz, FC2, and FC4 were used for generating VEPs in the frontal area; electrodes P5, P3, P1, Pz, P2, P4, and P6 for the parietal area; electrodes O1, Oz, and O2 for the occipital area; electrodes T8 and TP8 for the left temporal area; and electrodes T7, and TP7 for the right temporal area.

##### Disparity-Related Analysis

As shown in Figure 1B, the stimulus disparity settings include crossed disparities $-0.3^{\circ }$, $-0.6^{\circ }$, $-0.9^{\circ }$, $-1.1^{\circ }$, $-1.3^{\circ }$, and uncrossed disparities $0.3^{\circ }$, $0.6^{\circ }$, $0.9^{\circ }$, $1.1^{\circ }$, $1.3^{\circ }$. According to the study by Shibata et al. ([32]), disparities of $-0.6^{\circ }$, $-0.3^{\circ }$, $0.3^{\circ }$, $0.6^{\circ }$ were considered as the disparities within the comfort fusion zone (CFZ), and disparities of $-1.3^{\circ }$, $-1.1^{\circ }$, $1.1^{\circ }$, $1.3^{\circ }$ were considered as the disparities outside CFZ. Comparative analysis was performed for VEPs of each of the two disparity groups (crossed disparities versus uncrossed disparities; disparities in CFZ versus disparities outside CFZ) to obtain the disparity-related VEPs components. The individual EEG epochs from each identified disparity were first averaged to form the generated VEPs. Then, point-by-point statistics were adopted to identify the significant time range which differentiated various disparity groups. A cluster corrected method for multiple comparisons was adopted to control type 1 errors, where only when at least five consecutive sampling points (about 20 ms) return significant results (p<0.05 after correction) are the effects considered significant [23,33]. The peaks located within the significant time window were then considered as significant components relevant to disparity processing.

##### Block-Related Analysis

The four blocks were considered to be four SVF levels. Comparative analysis of VEPs in different SVF levels was performed to obtain the SVF-related VEP components under different disparities (crossed disparities, uncrossed disparities, disparities in CFZ, and disparities outside CFZ). The specific steps of VEPs generation were similar. Point-by-point statistics were adopted to identify the significant time window which differentiated various blocks in the corresponding disparities with 14 participants × 4 blocks. The peaks located within the significant time window were then considered significant components relevant to SVF.

#### 2.5.3. EEG cortical Source Localization

For each participant and IC, the DIPFIT toolbox in EEGLAB was used to estimate an equivalent current dipole located within a standardized three-shell boundary element head model based on the Montreal Neurological Institute (MNI) standard brain [34]. Principal component analysis (PCA) summed and compressed the dipole location and orientation by resulting in a 10-dimensional vector, then the neural ICs from all 14 participants were clustered with a k-means clustering algorithm (k = 18) in EEGLAB. ICs further than three standard deviations from any of the resulting cluster centroids were identified as an outlier cluster and subsequently eliminated from analysis. Clusters that contain less than half of the participants and the containing VEPs with component amplitudes below 0.1 μV were excluded, following previous studies [35,36].

## 3. Results

### 3.1. Subjective SVF Evaluation

**Figure 3:**
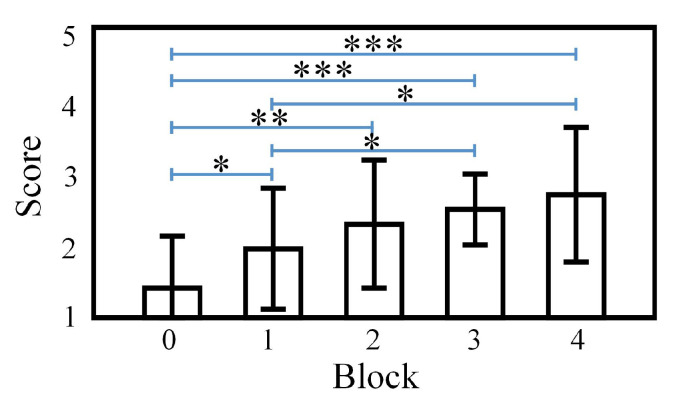
Statistical results of SVF scores for each viewing block. Block 0 refers to the assessment of SVF right before the formal stage begins. *** p<0.001, ** p<0.01, * p<0.05.

This study followed a repeated measures design on the same variable in four time periods (four blocks) for the same participants. The measurement time periods (Block 1, Block 2, Block 3 and Block 4) were the within-subject factors; the fatigue scores of each subject were the dependent variable. One-way repeated-measures ANOVA was performed on the visual fatigue scores, and the results are shown in Figure 3. Subjectively perceived SVF increased reliably among blocks, as a significant main effect was found for the factor block (F(4,52)=8.735, p<0.0001). Post hoc tests showed a steady increase in SVF ratings over the pre-experimental stage within the post-experimental blocks after Fisher’s LSD (least significant difference) corrected, and significant increases were observed between the pre-experimental stage and Block 1 (p=0.04), Block 2 (p=0.002), Block 3 (p<0.0001), Block 4 (p=0.0001), respectively. Multiple comparisons between post-experimental blocks indicated significant changes in SVF between Block 1 and Block 3 (p=0.014), as well as Block 1 and Block 4 (p=0.035).

### 3.2. Results of the Behavioral Task

After rejecting the null hypothesis of data Gaussianity using a Kolmogorov–Smirnov test (significant α=0.05), a non-parametric one-way analysis of variance (Friedman test) was performed on both the reaction time and accuracy followed by a Bonferroni post-hoc comparison. The mean and standard deviation of the reaction time in each block are as follows: Block 1 (803.4,135.4), Block 2 (795.2,156.0), Block 3 (782.7,146.7), and Block 4 (775.8,151.9). The results of the reaction time showed a significant decrease ($\chi^{2}\left(3\right)=20.3,p<0.001$) across the four viewing blocks. Multi-comparison revealed that significant differences were found between the following blocks (Block 1 and Block 4: p<0.001, Block 2 and Block 4: p<0.01, and Block 3 and Block 4: p<0.05). The mean and standard deviation of the correct accuracies in each block are as follows: Block 1 (96.28%,2.53), Block 2 (98.58%,1.33), Block 3 (96.58%,1.86), and Block 4 (97.72%,1.46). Reaction accuracies across all blocks did not show significant difference ($\chi^{2}\left(3\right)=6.54,p>0.05$).

### 3.3. VEP Characteristics with Different Disparities

Peaks N1, P2 and N2 were observed by the present study in each individual at the frontal area, while C1, P1, N1 and P2 were observed in each individual at the parietal area and occipital area. The latencies were 90 ms for negative C1 at the medial parietal area, 120 ms for P1 (P120), 120 ms for N1 at the frontal area (N120), 170 ms for N1 at the parietal area (N170), 200 ms for N1 at the occipital area (N200), 200 ms for anterior P2 (P200), 260 ms for anterior N2 (N260), and 260 ms for posterior P2 (P260).

**Figure 4:**
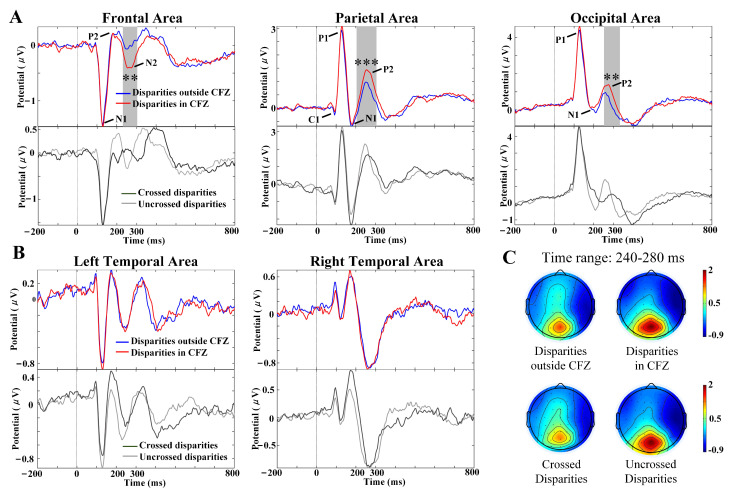
Effects of different disparities on VEPs components. (**A**) VEPs corresponding heat map and waveform images at frontal, parietal and occipital areas for crossed disparities versus uncrossed disparities, and disparities in CFZ versus disparities outside CFZ. (**B**) VEPs corresponding heat map and waveform images at left and right temporal areas for different disparities. (**C**) The topographical varieties of components P2 (time range: 240–280 ms) in the 2 disparity groups. The grey underlining denotes time region of amplitude significant differences after the cluster method corrected, *** p<0.001, ** p<0.01.

As shown in Figure 4A,B, point-by-point statistical analysis of VEPs evoked by crossed disparities versus uncrossed disparities, and disparities in CFZ versus disparities outside CFZ, revealed that only one significant time interval (marked as gray underlining in Figure 4A, multiple comparisons corrected by the cluster method) ranging from 240 ms to 280 ms existed at most sites in the frontal, parietal and occipital areas, was associated with disparities in CFZ versus disparities outside CFZ in the VEPs. The VEP waveforms showed that the amplitudes of component N2 evoked by disparities in CFZ were significantly greater in the frontal area (p=0.002, time range: 220–304 ms), and those of component P2 in the parietal (p=0.0007, time range: 204–304 ms) and occipital (p=0.009, time range: 252–320 ms) areas were greater than those evoked by disparities outside CFZ. The results of posterior P2 characteristics with one-way repeated-measures ANOVA analysis for Pz, POz and Oz electrodes are formed in Table 1, where the peak amplitudes of component P2 evoked by uncrossed disparities were significantly greater than the crossed disparities, while latencies evoked by uncrossed disparities were significantly earlier than the crossed disparities. Figure 4C showed the averaged topography of four disparity groups in time ranging from 240 ms to 280 ms, in which component P2 evoked by disparities in CFZ and uncrossed disparities had greater peak amplitudes in the parietal–occipital area than those of component P2 evoked by disparities outside CFZ and crossed disparities, respectively. Further, repeated-measures ANOVA analysis was conducted to analyze the component P2 characteristics of Pz, POz and Oz electrodes between uncrossed disparities outside CFZ, uncrossed disparities in CFZ, crossed disparities in CFZ, and crossed disparities outside CFZ, as shown in Table 2. All *p* values were adjusted with the Greenhouse–Geisser epsilon correction for nonsphericity if necessary. Multiple comparisons with Bonferroni corrected indicated that peak amplitudes and latencies of channel POz and Oz showed significant differences between uncrossed disparities in CFZ and crossed disparities outside CFZ.

### 3.4. SVF Effects on Scalp VEP Components

**Figure 5:**
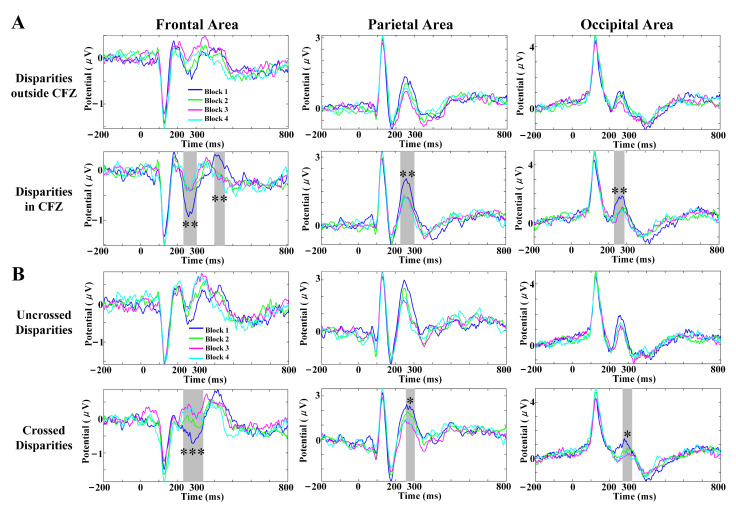
Effects of SVF levels among different disparities on VEPs components. (**A**) VEPs waveform images at frontal, parietal and occipital areas of 4 blocks for disparities outside CFZ and disparities in CFZ, respectively. (**B**) VEPs waveform images at frontal, parietal and occipital areas of 4 blocks for uncrossed disparities and crossed disparities, respectively. The grey underlining denote time region of peak amplitude significant difference after the cluster method corrected, *** p<0.001, ** p<0.01, * p<0.05.

Figure 5 demonstrates the characteristics of VEPs in the frontal, parietal and occipital areas with several SVF levels induced by disparity variation under different disparities. As shown in Figure 5A, the peak amplitudes of disparities in CFZ induced SVF had a significant difference in the frontal (p=0.002, time range: 236–304 ms; p=0.004, time range: 400–444 ms), parietal (p=0.003, time range: 232–304 ms), and occipital (p=0.002, time range: 236–288 ms) areas (marked with gray underlining, multiple comparisons corrected by the cluster method), which were mainly due to the sharp decrease in amplitudes from Block 1 to the remaining blocks. In Figure 5B, the peak amplitudes of crossed disparities induced SVF show significant difference in the frontal (p=0.0002, time range: 236–340 ms), parietal (p=0.037, time range: 260–304 ms), and occipital (p=0.025, time range: 276–320 ms) areas (marked with gray underlining, multiple comparisons corrected by the cluster method). The peak amplitudes of component P2 with disparities outside CFZ, crossed disparity and uncrossed disparity induced SVF showed a similar monotonously decreasing trend along with the increased levels of SVF (block 1–block 3).

### 3.5. IC Clusters

**Figure 6:**
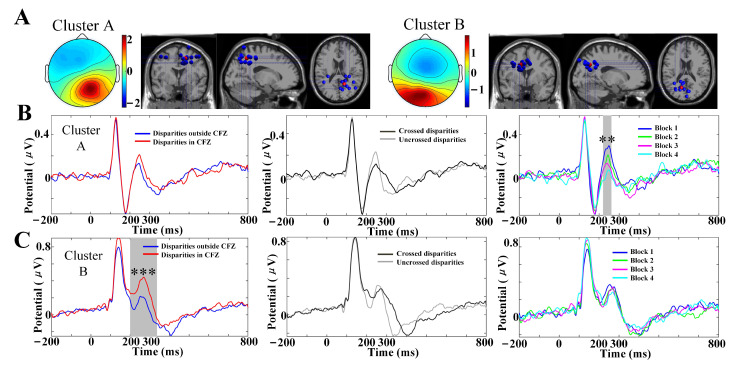
(**A**) Scalp maps and dipole locations of IC clusters contained component P2. Each cluster is visualized in the MNI brain volume in coronal, sagittal, and top views. Blue-colored spheres indicate the dipole locations for single cortical sources for single participants, and red-colored spheres indicate cluster centroids. (**B**) VEPs comparison results of cluster A. (**C**) VEPs comparison results of cluster B. The grey underlining denotes time region of peak amplitude significant differences after the cluster method corrected, *** p<0.001, ** p<0.01.

Two clusters containing posterior P2 were located in the right parieto-occipital cortices (cluster A, 13 participants, 14 ICs) and left parieto-occipital cortices (cluster B, 10 participants, 14 ICs). Figure 6A shows dipole locations of the clustered ICs and centroids visualized in the MNI brain volume. Additionally, Table 3 displays the coordinates of cluster centroids and the number of participants and sources contained in each cluster. According to the MNI coordinates, both cluster A and cluster B were located in the medial part of the Brodmann area 7 (BA 7), which is also known as the precuneus. Point-by-point repeated-measurement ANOVA was conducted on the VEPs of the two clusters, as shown in Figure 6B,C: sample points of 220–260 ms time interval in cluster A and sample points of 204–336 ms in cluster B, which could be viewed as component P2, varied significantly with blocks (p=0.008, cluster method corrected) and disparity conditions with CFZ (p=0.0002, cluster method corrected), respectively. The waveform of VEPs in cluster A was quite similar with that in the parietal area (shown in Figure 4A). These findings imply that the VEPs observed in parietal areas are mainly attributed to activities from the precuneus.

## 4. Discussion

This paper proposed a Go/NoGo paradigm based on disparity variations to induce SVF and explored the effects of disparity variations as well as SVF on the temporal characteristics of VEPs while maintaining the attention to the viewing tasks. Point-by-point permutation statistical results and repeated measures ANOVA results revealed that the peak amplitudes of sample points within the time window from 240 ms to 280 ms at the parietal and occipital brain areas changed significantly along with the different disparities and SVF levels, which indicated that posterior P2 of VEPs may be related to the depth perception in stereo vision, and thus related to SVF induced by disparity variations.

The subjective results demonstrated that there was a significant increase in SVF levels at each post-experimental stage compared with those of the pre-experimental stage, and the SVF levels of the subjects gradually increased after each block as the experimental phase progressed. Therefore, according to the subjective evaluation, the paradigm proposed in this paper did induce SVF, and levels of the induced SVF gradually increased with the extension of the experimental viewing time. In addition, the accuracy of the judgments and response time of the four blocks indicated that the subjects maintained a certain level of attention and did not suffer a severe mental process during our experiment.

Components P1, N1, P2, and N2 of VEPs were observed in the present study, which may reflect the stereoscopic depth perception process related to binocular disparity. Our findings showed that stereoscopic depth perception with the stimuli of disparity in CFZ significantly increased both the anterior N2 and posterior P2 peak amplitudes compared with the stimuli of disparity outside CFZ (shown in Figure 4), while stereoscopic depth perception with convergence stimuli both significantly decreased the posterior P2 peak amplitudes and significantly delayed posterior P2 latencies compared with the divergence stimuli (shown in Table 1 and Table 2). In the current studies, the frontocentral negative wave around 200–400 ms in visual tasks after stimulus onset (N2) in NoGo trials was interpreted as reflections (or an outcome) of inhibitory processes in the frontal cortex [37], suggesting that the anterior N2 is associated with cognitive control [38]. Our findings also apply to this conclusion. NoGo N2 was reported to be larger in participants with low rather than high false alarm rates, suggesting an association between the amplitude and successful response inhibition [39]. From this perspective, compared with disparities outside CFZ, disparities in CFZ were easier for fusion recognition and thus elicited larger N2 amplitudes. Alternatively, the decrease in anterior N2 amplitudes with the levels of SVF among different disparities in Figure 5 could be explained by the suppression of cognitive activities when SVF occurred. The parieto-occipital P2 is involved in cognitive processes, such as memory performance [40] and working memory [41]. Furthermore, studies have shown that component P2 is supposed to be involved in extracting form from motion [42] and in recognizing the motion of objects in the visual field [43]. However, the mechanism of modulating P2 during stereoscopic vision is not clear, as few previous reports on P2 potentials examined 3D depth perception. P2 was reported to be both elicited by stereo and non-stereoscopic contents in the parietal and occipital lobes [20], and characterize the initial perception and recognition of 3D objects [21]. The occipital P2 was found to have a mid-relevance to the disparity [44] and sensitivity to both the magnitude and the direction of the disparity [17,18]. All the above studies strongly support our observation that posterior P2 in our study could be related to depth perception.

The occipital and parietal regions are deeply involved in depth perception. Patients with occipital/parietal lesions were unable to see any depth in RDSs [45]. Moreover, studies have shown that perception and neuronal activity have a strong connection in the extrastriate cortex and the primary visual cortex [46]. Disparity-selective neurons can be found in extrastriate areas as well as in visuomotor regions of the parietal and frontal cortex, suggesting the widespread use of binocular signals [46,47,48,49,50]. Tuned near and far cells give maximal responses at crossed or uncrossed disparities, respectively, while near and far cells have reciprocal disparity tuning functions, being activated over a wide range of crossed or uncrossed disparities respectively and suppressed by the opposite disparity [47]. Ref. [51] found that there was change in the VEP amplitude between the large and small disparity responses, which was consistent with the existence of rather separate fine and coarse mechanisms. Thus, we speculate that the difference of posterior P2 characteristics in our results under different disparities may be related to the function of these disparity-selective neurons, which still need further validation.

The task settings of the Go/NoGo paradigm in our study were in line with the top-down attentional process. Top-down deployment of visual–spatial attention is conveyed by cortical feedback connections from the frontal regions to lower sensory areas, the magnitude of top-down modulation on neuronal firing tends to increase across the cortical hierarchy, and both the prefrontal cortex (PFC) areas and posterior parietal cortex (PPC) areas can provide top-down signals to control attention [52,53,54]. Thus the anterior N2 and posterior P2 components observed around 260 ms in the experiment may also be involved in the modulation of the top-down attentional process. During top-down attention, attention is oriented to the location with the most prominent activity, and signals to generate an eye movement are delivered accordingly through the superior colliculus (SC) [53], while PPC has been shown to be the target of output from SC [55], which suggests that posterior P2 may be associated with eye movements in binocular disparity-based depth perception. The decrease trend in posterior P2 could also indicate the attentional inhibition after SVF.

The eye vergence is the opposite movement of both eyes. It changes the binocular disparity and is driven by the depth changes of a target object. Studies have reported that the strength and time of eye vergence coincided with the onset and strength of the VEPs during the deployment of top-down attention [54]. In our study, latencies of posterior P2 coincided with the timing of depth perception-related vergence due to disparity changes. Vergence latency requires about 180–250 ms for convergence and 200–210 ms for divergence, while vergence requires about 195–750 ms for convergence and 240–1000 ms for divergence, which demonstrates asymmetric behavior [56,57]. The same asymmetry was also found in our results (shown in Table 1 and Table 2), as posterior P2 elicited by crossed disparities (275–290 ms) had a longer latency compared with uncrossed disparities (260–270 ms). In contrast, no difference in posterior P2 latency was observed between disparities in CFZ and disparities outside CFZ, possibly because each disparity group included both crossed and uncrossed disparities, and the disparity settings (minimum of $\pm 0.3^{\circ }$, maximum of $\pm 1.3^{\circ }$) in VR environment was not enough to lead to differences in the vergence time due to the difference between large and small disparities. Therefore, our results further suggest that posterior P2 could be related to depth perception, reflecting the process of the brain’s response to vergence during depth perception.

SVF factor was reflected in posterior P2 of VEPs through point-by-point statistics (shown in Figure 5), in which the peak amplitudes of posterior P2 tended to decrease monotonically with increasing SVF among almost all disparities. Although the neural mechanisms of SVF still remain unrevealed, some studies suggest that fatigue is reflected in sensory perception and self-awareness [58,59]. With this understanding, the decrease in performance of the peripheral system is actually caused by the inhibition of the central system, which creates a sense of fatigue based on the input from the peripheral system [58]. SVF may follow the same pattern.

The source localization results showed that posterior P2 was located in the precuneus (shown in Table 3) since a high similarity of VEP waveforms was found between the precuneus from cluster-based analysis and the parietal area from scalp analysis (shown in Figure 4 and Figure 6). Thus, the precuneus might be one of main generators of VEP components C1, P1, N1 and P2 observed in parietal–occipital lobes. The medial aspect of the PPC has historically been referred to as the precuneus, and the territory of the precuneus mainly corresponds to the mesial extent of BA 7, which also occupies most of the lateral parietal cortex above the intraparietal sulcus [60]. The precuneus is located at the middle part of the dorsal visual pathway, which supports both conscious and non-conscious visuospatial processing. It received the transformed retinotopic representation from the occipito-parietal circuit and then passed the representation of the egocentric depth to the following parieto-temporal, parieto-prefrontal, and parieto-middle temporal pathways [61,62]. Recent functional imaging findings suggest a central role for the precuneus in a wide spectrum of highly integrated tasks, including egocentric depth perception, visuo-spatial imagery, self-consciousness, spatial navigation and affective response to pain [60,63]. Therefore, decreased peak amplitudes of posterior P2 among SVF levels might represent an inhibition of neural activities related to egocentric location awareness, which could also be seen as an inhibition of the ability related to depth perception.

Generally, this study investigated the ERPs formed by different disparities and stereoscopic visual fatigue under the stereoscopic display device HMD. For the next step, visual fatigue caused by different types of displays, especially stereoscopic displays and non-stereoscopic displays, will be considered. HMD, 3D TV, and 2D display will all be used as display devices, and the ERPs components of visual fatigue under the three types of displays will be compared, so as to obtain the ERPs components related to stereoscopic visual fatigue (HMD/3D TV) and non-stereoscopic visual fatigue (2D display). There still exist limitations in the current study. VEPs have high temporal resolution, making them the ideal method to study brain information processing. In this study, a relatively large number of electrode channels were selected to acquire EEG signals to alleviate the lack of spatial resolution, but the desired spatial accuracy may not be achieved when performing source localization compared to fMRI. VEPs combined with fMRI would be a potential method to study SVF in further research.

## 5. Conclusions

In this paper, we designed a Go/NoGo paradigm for attention sustaining, and constructed an experimental scenario of SVF induced by disparity variations in the VR environment for the first time. RDSs were presented as visual stimuli, and the temporal characteristics of VEPs elicited by disparity changes were obtained. Point-by-point statistics and one-way repeated measures ANOVA were adopted to explore the relationship between disparities/SVF and VEP component characteristics. In our study, posterior VEP components, such as C1 (about 90 ms), P1 (about 120 ms), N1 (about 170 ms), and P2 (about 260 ms) elicited by disparity variations, were observed, which demonstrated that posterior P2 originated from the precuneus could be related to depth perception, reflecting the process of the brain’s response to vergence during depth perception, and thus related to the levels of SVF induced by disparity variations. In conclusion, VEP posterior P2 has the potential to be an indicator of disparity variation that distinguishes comfort from discomfort in VR content as well as being an effective indicator for evaluating SVF.

## Figures and Tables

**Table 1:** Statistical results of component P2 characteristics with crossed and uncrossed disparities at channel Pz, POz and Oz.

Channel	Disparity	Peak Amplitude (μV)	Latency (ms)	Repeated-Measures ANOVA Analysis (Peak Amplitude)	Repeated-Measures ANOVA Analysis (Latency)
Pz	Crossed	1.848 (1.744)	275.89 (28.92)	*F*(1,69) = 3.531;	*F*(1,69) = 7.944;
	Uncrossed	2.064 (1.743)	266.83 (22.98)	*p* = 0.0645	*p* = 0.006
POz	Crossed	2.567 (1.909)	278.91 (30.52)	*F*(1,69) = 8.317;	*F*(1,69) = 19.802;
	Uncrossed	3.113 (2.119)	263.43 (19.51)	*p* = 0.005	*p* < 0.0001
Oz	Crossed	1.989 (1.870)	290.80 (32.46)	*F*(1,69) = 12.312;	*F*(1,69) = 39.133;
	Uncrossed	2.533 (1.709)	266.06 (23.32)	*p* = 0.0008	*p* < 0.0001

**Table 2:** Statistical results of component P2 characteristics with different disparities at channel Pz, POz and Oz.

Channel	Disparity	Peak Amplitude (μV)	Latency (ms)	Repeated-Measures ANOVA Analysis (Peak Amplitude)	Repeated-Measures ANOVA Analysis (Latency)	Multiple Comparisons (Peak Amplitude)	Multiple Comparisons (Latency)
Pz	Uncrossed outside CFZ	2.029 (1.913)	273.29 (30.66)				
	Uncrossed in CFZ	2.108 (1.704)	266.71 (21.17)	*F*(3,81) = 1.287;	*F*(3,81) = 1.272;		
	Crossed in CFZ	1.978 (1.775)	270.71 (27.09)	*p* = 0.285	*p* = 0.289		
	Crossed outside CFZ	1.765 (1.874)	276.43 (29.92)				
POz	Uncrossed outside CFZ	3.128 (2.291)	267.57 (25.45)				
	Uncrossed in CFZ	3.203 (1.962)	261.00 (13.18)	*F*(2.127,57.441) = 3.697;	*F*(1.923,51.921) = 4.965;	*p*(B,D) = 0.038	*p*(A,C) = 0.057
Crossed in CFZ	2.838 (1.879)	278.29 (22.90)	*p* = 0.029	*p* = 0.012		*p*(B,C) = 0.0004	
	Crossed outside CFZ	2.389 (1.940)	277.29 (37.56)				
Oz	Uncrossed outside CFZ	2.477 (1.536)	270.86 (15.55)				*p*(A,C) = 0.002
	Uncrossed in CFZ	2.641 (1.899)	264.57 (31.17)	*F*(3,81) = 4.376;	*F*(2.376,64.164) = 7.336;	*p*(A,D) = 0.051	*p*(B,C) = 0.006
	Crossed in CFZ	2.138 (1.666)	289.57 (24.01)	*p* = 0.007	*p* = 0.0007	*p*(B,D) = 0.055	*p*(B,D) = 0.020
	Crossed outside CFZ	1.817 (2.419)	290.29 (41.80)				

Notes: A represents uncrossed disparities outside CFZ, B represents uncrossed disparities in CFZ, C represents crossed disparities in CFZ, and D represents crossed disparities outside CFZ. Only significant results close to or below 0.05 are shown in the table.

**Table 3:** Clusters of independent sources contained posterior component P2 obtained with ICA.

Cluster	Location of Cluster Centroid (Brodmann Area)	Cluster Centroid Coordinates (x, y, z)	Number of Participants (Ps) and Ics	VEPs Components
A	Precuneus (BA7)	12, −53, 51	13 Ps, 14 ICs	C1, P1, N1, P2
B	Precuneus (BA7)	−14, −67, 37	10 Ps, 14 ICs	C1, P1, N1, P2

## Data Availability

Not applicable.
